# Mathematical Models for Sleep-Wake Dynamics: Comparison of the Two-Process Model and a Mutual Inhibition Neuronal Model

**DOI:** 10.1371/journal.pone.0103877

**Published:** 2014-08-01

**Authors:** Anne C. Skeldon, Derk-Jan Dijk, Gianne Derks

**Affiliations:** 1 Department of Mathematics, University of Surrey, Guildford, Surrey, United Kingdom; 2 Faculty of Health and Medical Sciences, University of Surrey, Guildford, Surrey, United Kingdom; Fondazione Edmund Mach, Research and Innovation Centre, Italy

## Abstract

Sleep is essential for the maintenance of the brain and the body, yet many features of sleep are poorly understood and mathematical models are an important tool for probing proposed biological mechanisms. The most well-known mathematical model of sleep regulation, the two-process model, models the sleep-wake cycle by two oscillators: a circadian oscillator and a homeostatic oscillator. An alternative, more recent, model considers the mutual inhibition of sleep promoting neurons and the ascending arousal system regulated by homeostatic and circadian processes. Here we show there are fundamental similarities between these two models. The implications are illustrated with two important sleep-wake phenomena. Firstly, we show that in the two-process model, transitions between different numbers of daily sleep episodes can be classified as grazing bifurcations. This provides the theoretical underpinning for numerical results showing that the sleep patterns of many mammals can be explained by the mutual inhibition model. Secondly, we show that when sleep deprivation disrupts the sleep-wake cycle, ostensibly different measures of sleepiness in the two models are closely related. The demonstration of the mathematical similarities of the two models is valuable because not only does it allow some features of the two-process model to be interpreted physiologically but it also means that knowledge gained from study of the two-process model can be used to inform understanding of the behaviour of the mutual inhibition model. This is important because the mutual inhibition model and its extensions are increasingly being used as a tool to understand a diverse range of sleep-wake phenomena such as the design of optimal shift-patterns, yet the values it uses for parameters associated with the circadian and homeostatic processes are very different from those that have been experimentally measured in the context of the two-process model.

## Introduction

Reduced or mis-timed sleep is increasingly recognized as presenting a significant health risk and has been correlated with increases in a diverse range of medical problems including all-cause mortality, cardio-vascular disease, diabetes and impaired vigilance and cognition [1]–[5]. The biological mechanisms that result in such problems are beginning to be understood: recent work has shown that changes to the duration or timing of the human sleep-wake cycle can result in the up- or down regulation and changes to the temporal pattern of large numbers of genes associated with biological processes including metabolic, inflammatory, immune, stress responses and circadian rhythmicity [6], [ 7]. To further understand the underlying phenomena and associations that govern sleep-wake regulation, mathematical models are an important tool to help clarify concepts, challenge accepted ideas and aid in the interpretation of data.

A review of early mathematical models of sleep is given in [8], leading up to the seminal model of Borbély, Daan and Beersma [9], [ 10], usually called the two-process model, and extended by Borbély and Achermann [11]. As indicated by its name, the two-process model proposes that the sleep-wake cycle can be understood in terms of two processes: a homeostatic process and a circadian process. The homeostatic process takes the form of a relaxation oscillator that results in a monotonically increasing ‘sleep pressure’ during wake that is dissipated during sleep. Switching from wake to sleep and from sleep to wake occurs at upper and lower threshold values of the sleep pressure respectively, where the thresholds are modulated by an approximately sinusoidal circadian oscillator. This model has proved compelling for both its physiological grounding and its graphical simplicity and has been used extensively (there are over 1500 citations to [9] and 600 citations to [10] to-date). For example: to explain why only a relatively short period of recovery sleep is needed to compensate for even lengthy periods of sleep deprivation [9]; to explain chronotype changes in adolescents [12]. Extensions of the two-process model have been developed to explain the results of chronic sleep restriction experiments [13], [ 14]. Despite its success, it remains difficult to relate the threshold values in the two-process model and its extensions to physiological processes.

Advances in neurophysiology have led to a proliferation of models that aim to extend the two-process model to a more physiological setting [15]–[21]. A recent review is given in [22]. The most extensively tested of these is the model of Phillips and Robinson [17] (the PR model), which has been used to explain sleep fragmentation experiments [23], differences in mammalian sleep patterns [24] and subjective fatigue during sleep deprivation [25]. The PR model has also been extended to allow for the inclusion of the effects of caffeine [26] and to allow for feedback of the sleep-wake cycle on the circadian oscillator in order to explain spontaneous internal desynchrony [27], [ 28].

In [17], [ 20], it was observed that the results of two different physiologically based models could be presented in a qualitatively similar way to those from the two-process model. Here we show that some features of the PR model are not only qualitatively, but also quantitatively similar to the two-process model: the parameters in the PR model can be explicitly related to the parameters in the two-process model, giving a physiological interpretation to the thresholds in the two-process model. We illustrate the consequences of this explicit relation with two important sleep-wake phenomena. First, by using the fact that the two-process model can be represented as a one-dimensional map with discontinuities [29], [ 30], we demonstrate how transitions between monophasic and polyphasic sleep occur through grazing bifurcations. These grazing bifurcations are then used to provide a theoretical underpinning for the observations that many mammalian sleep patterns can be understood within a common framework by varying just two parameters in the PR model [24]. Second, turning to sleep deprivation experiments, we show how the ‘wake effort’ concept introduced in the PR model to explain sleep deprivation can be explicitly related to the two-process model. This shows that the wake effort is closely related to the difference between the homeostatic pressure and the circadian oscillator, a measure often used in the context of the two-process model to understand sleepiness. Furthermore we discuss briefly how the PR model may explain effects of chronic partial sleep deprivation on waking performance.

## Results

First we give a summary of the main features of the two-process model and the PR model.

### The two process model

The two-process model considers a homeostatic pressure 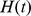 that decreases exponentially during sleep, 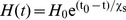
(1)and increases during wake, 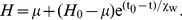
(2)


The parameter 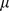 is known as the ‘upper asymptote’ [13], [ 14], this is the value that the homeostatic pressure 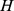 would reach if no switch to sleep occurred. Similarly there is a ‘lower asymptote’ of zero. Switching between wake and sleep occurs when the homeostatic pressure 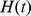 reaches an upper threshold, 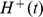, that consists of a mean value 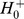 modulated by a circadian process 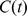, 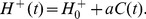
(3)


**Figure 1:**
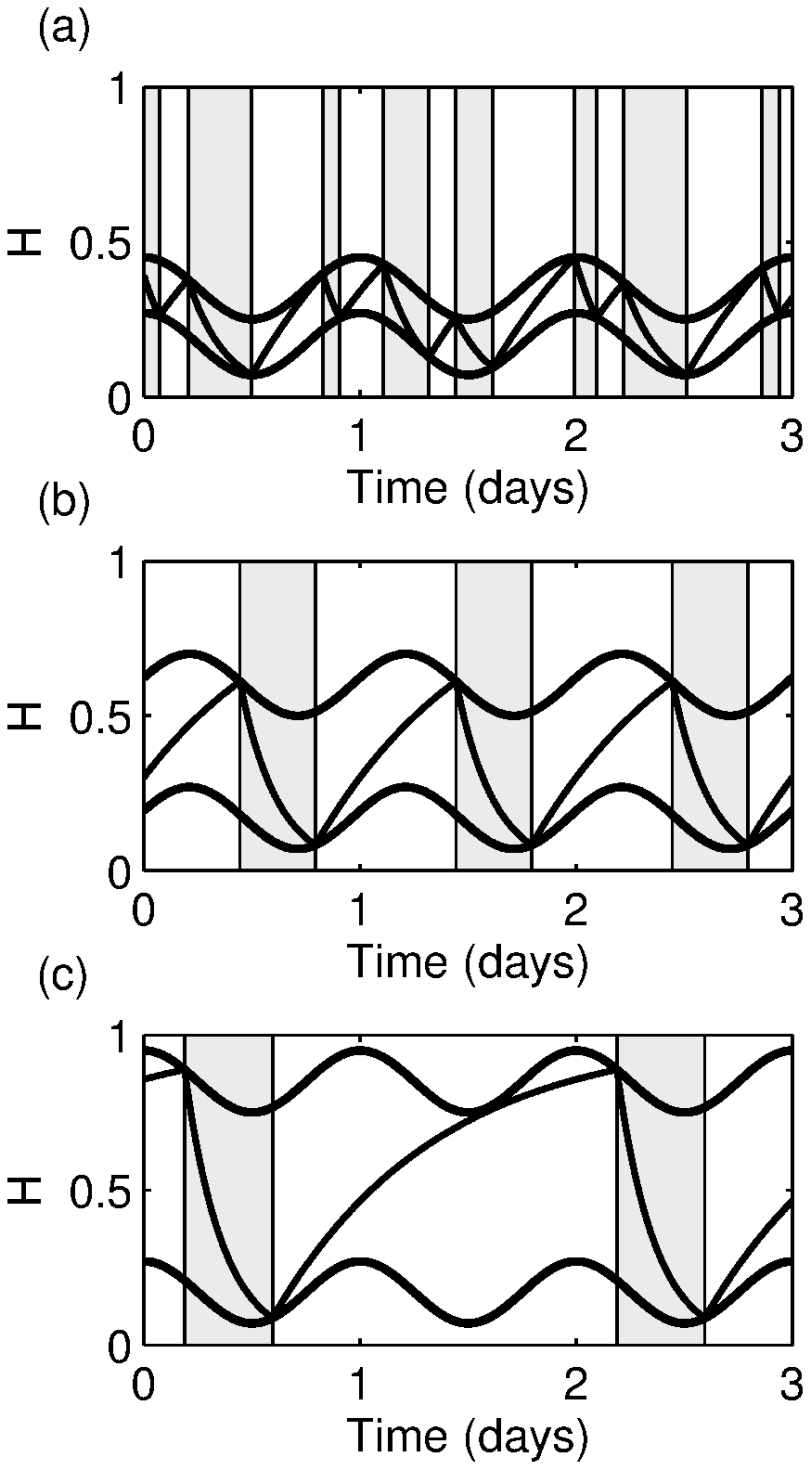
Sleep-wake cycles generated by the two-process model. With the parameters as in [10], Figure 3: 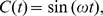 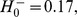 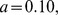 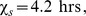 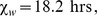 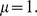 (a) 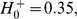 (b) 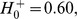 (c) 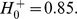 The times when sleep occurs (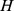 decreasing) are shaded.

The switch between sleep and wake occurs when 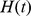 reaches a lower threshold, 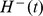, 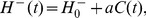
(4)where 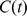 is a periodic function of period 24 hours. In the simplest cases 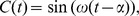
but more complicated forms that include higher harmonics, such as 
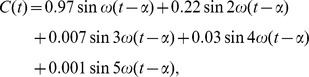
have also been used [11]. Typical results of this model illustrating its rich dynamics are shown in Figure 1.

### Phillips and Robinson model (PR model)

**Figure 2:**
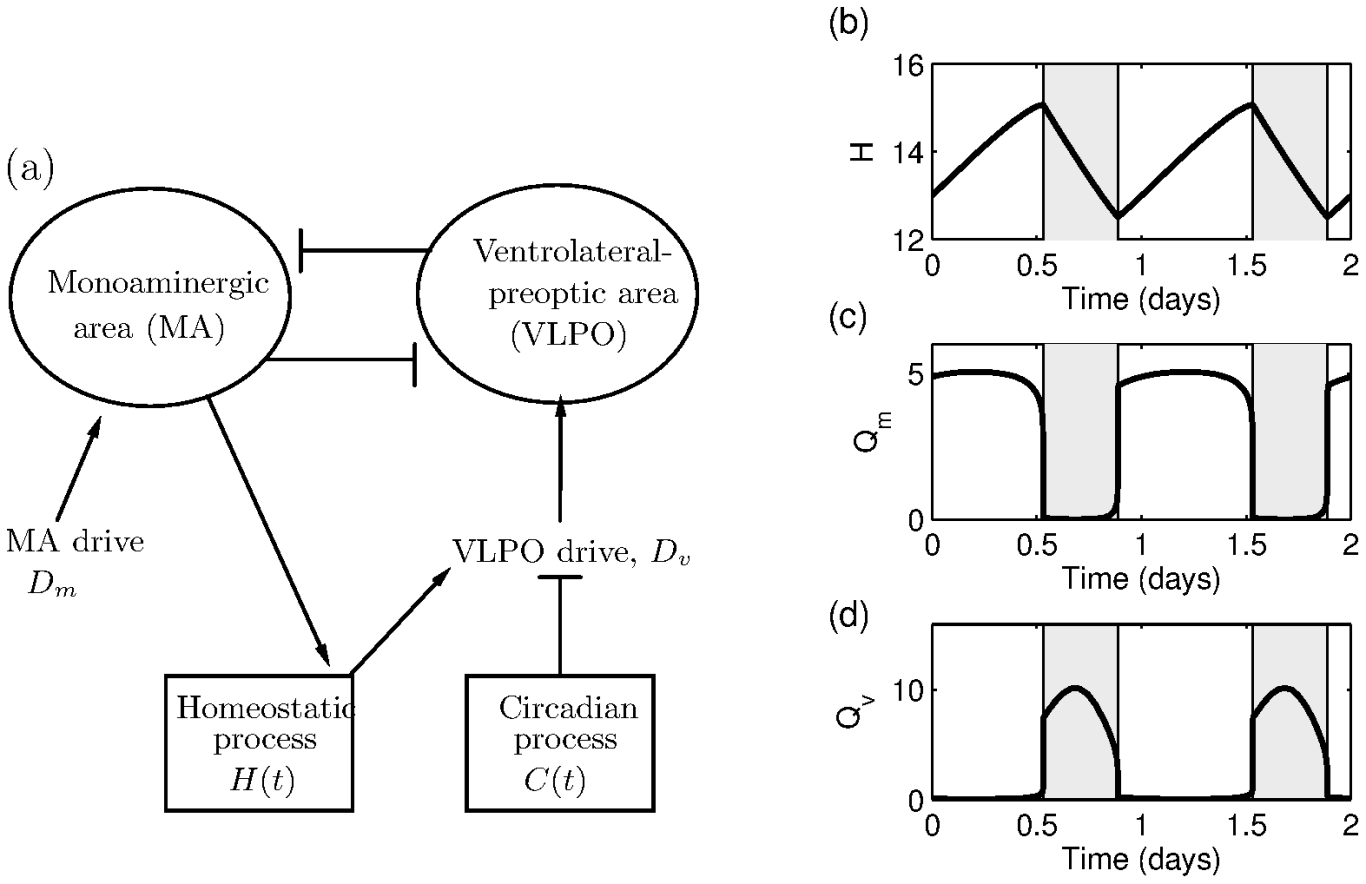
The PR model and typical solutions. (a) Diagrammatic description of the PR model showing the links between the VLPO, MA, the homeostatic and the circadian processes. (b), (c) and (d) show typical timeseries for the level of the homeostat, 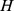, and the firing rates of the MA and VLPO, 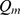 and 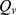, respectively. The times where sleep occurs are shaded.

At the core of the PR model are two groups of neurons: mono-aminergic (MA) neurons in the ascending arousal system that promote wake and neurons based in the ventro-lateral pre-optic (VLPO) area of the hypothalamus that promote sleep. Phillips and Robinson model the interaction between the MA and the VLPO as mutually inhibitory. In the absence of further effects, this would mean that the model would either stay in a state with the MA active (wake) or in a state with the VLPO active (sleep) and no switching between the states would occur. Switching between sleep and wake occurs because the model also includes a drive to the VLPO that is time dependent and consists of two components: a circadian drive, 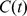, and a homeostatic drive 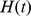. The structure of the PR model is shown in Figure 2(a).

The neurons are modelled at a population level and are represented by their mean cell body potential relative to rest, 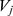 for 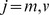, where 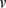 represents the VLPO group and 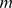 represents the MA. The potential is related to the firing rates of the neurons by the firing function, 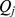, 
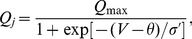
(5)where 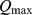 is the maximum firing rate and 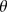 is the mean firing threshold relative to resting. The function 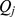 is a sigmoid function, which is close to zero for all negative values of 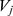 and then saturates exponentially fast to 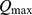.

The neuronal dynamics are represented by 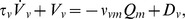


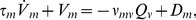
(6)where the drive to the VLPO, 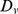 and to the MA, 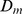 are given by 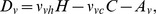


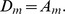



The homeostatic component of the drive, 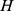 is modelled by 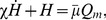
(7)and the circadian drive, 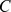, is approximated by 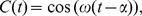
where 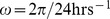 and 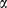 is a phase shift that specifies the distance from the circadian maximum. Typically, 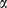 is chosen so that the switch from sleep to wake occurs at an appropriate clock time.

Typical results produced by the PR model are shown in Figure 2(b)–(d). During wake, the firing rate of the MA neurons is high (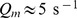), that of the VLPO is low and the homeostatic pressure tends to increase, while during sleep the firing rate of the MA neurons is low (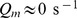), that of the VLPO is high and the homeostatic pressure tends to decrease. Note that in the PR model switching between wake and sleep is defined to occur when 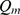 reaches the threshold value of one; this differs from the timing of the maximum and minimum homeostatic pressure by a few minutes. Obviously, the exact choice of the threshold value does not play an important role in the dynamics of the system, but does change the regions that are labelled as sleep or wake.

### Comparison of the PR and two-process models

As recognised in [23], since changes in neuronal potentials happen much faster than changes associated with the homeostatic pressure, 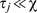, there is a strong separation of timescales in the PR model. This strong separation of timescales means that the dynamics of the PR model is well approximated by two separate models: one on the ‘slow’ timescale that is appropriate when considering changes on the timescale of the circadian and homeostatic processes such as the timings of sleep and wake; and the other, the ‘fast’ timescale, which is appropriate when considering changes on the timescale of the neuronal potentials such as the response to a night time disturbance. If the firing switching function 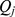 given in equation (5) in the PR model is replaced by a hard switch, 
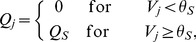
(8)where 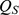 is the mean maximum firing rate of the neuronal population and 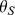 is the value at which the switch occurs, we show in the Methods section that the parameters for the slow dynamics of the PR model with a switch can be exactly mapped to parameter values in the two-process model, specifically, 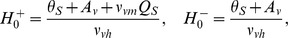


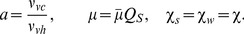
(9)


The lower threshold is therefore dependent on the mean drive to the VLPO and the threshold firing rate. The difference between the thresholds in the two-process model, 
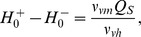
can then be interpreted physiologically as the amount by which the MA inhibits the firing of the VLPO during wake. This makes intuitive sense: there is hysteresis in the switch between wake and sleep because of the mutual inhibition between the MA and the VLPO. In the wake state, the VLPO requires a large drive to fire to counteract the inhibitory effects of the MA. Once in the sleep state, less drive is needed to maintain firing because the MA is quiescent.

**Figure 3:**
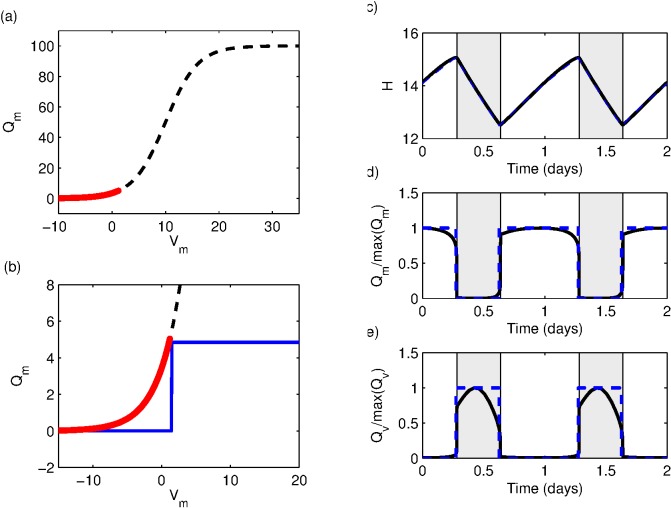
The PR model and the PR switch model. (a) The dashed (black) line shows the firing function given by equation (5); the thicker (red) line shows the portion that is used for the ‘normal’ PR cycle. (b) A magnified version of (a). The thin (blue) line shows the switch function (8). Panels (c),(d) and (e) show the behaviour of the homeostat, 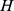, and the firing rates 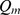 and 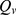 for the PR model (solid line) and the PR model with the hard switch (dashed line). The switch parameters are 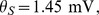 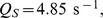 the mean firing rate of the neural population during wake; all other parameters are listed in the Tables section.

Using the standard parameters for the PR model, only a small part of the firing function (5) is used. This is illustrated in Figure 3(a), where the firing function is shown by the dashed line and the typical range of values for 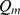 is shown by the thick (red) line. We show in the Methods section that there is a systematic way to relate the parameters for the original PR model to equivalent parameters for the two-process model that retain the timings and values at the extrema of the homeostat. In keeping with the fact that the mean firing rate across the neuronal population 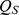 is much less than the maximum possible firing rate 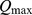, the value for 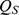 is significantly less than 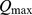 but close to the mean firing rate across the population in the PR model: in fact the actual firing function needed in the PR-switch model is shown by the blue line in Figure 3(b).

**Figure 4:**
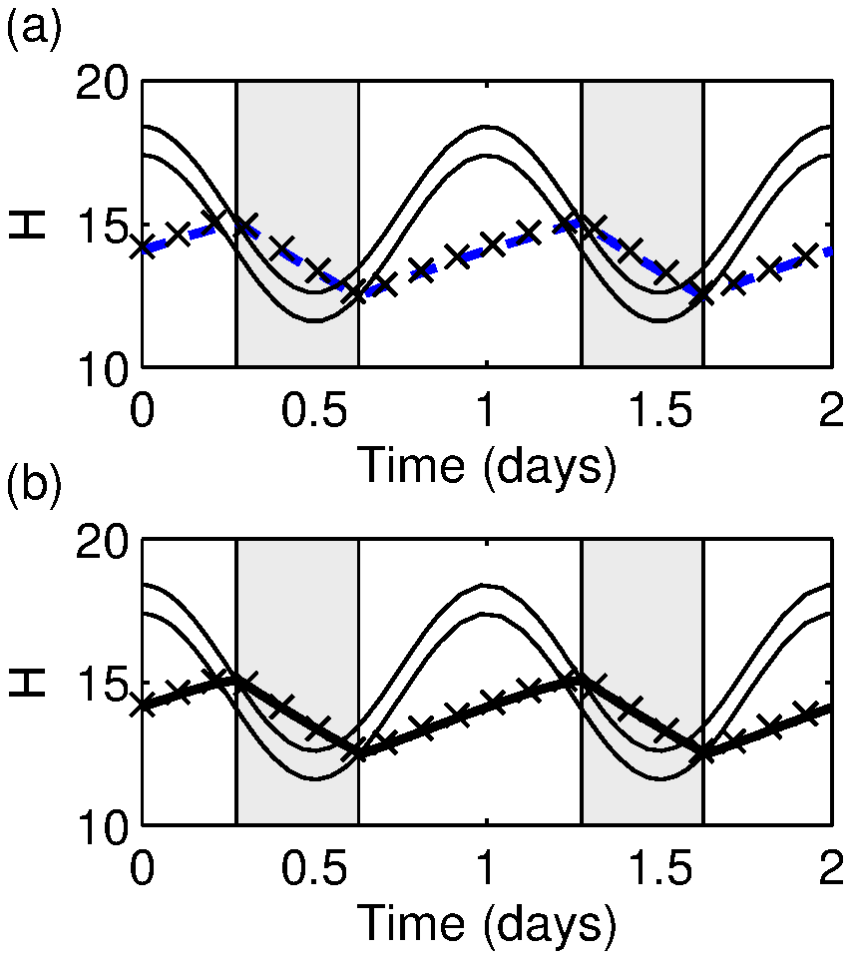
The two-process model compared to the two PR models. (a) Comparison of the PR switch model with the two-process model. (b) Comparison of the PR model with the two-process model. Crosses show the two-process model; solid line the PR model and (blue) dashed line the PR switch model.

Typical graphs of 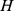 and 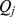 for both the original PR model and the PR switch model are shown in Figure 3(c)–(e) demonstrating the close agreement between the two cases. Graphs comparing timeseries computed from the two-process model and numerical integrations of the corresponding PR/PR switch model are shown in Figure 4. The extremely good agreement of the two models is a result of the very large disparity in timescales between the fast and slow systems. Consequently, solutions of the PR model converge to solutions on the slow manifold on the timescale of minutes. Once on the slow manifold, solving the PR model is essentially equivalent to solving the two-process model.

In [31] it was recognised that the PR model could be plotted in a similar way to the two-process model, but the explicit connection between parameters was not made. It is stated that a key difference is that in the two-process model the value of 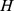 remains between the thresholds at all times, as in Figure 1. However, we note that this could be regarded as a matter of parameter choice rather than a fundamental difference between the two models: whether the two-process model remains between the thresholds depends on the relative gradients of the circadian and homeostatic processes at each wake/sleep or sleep/wake transition. Figure 4 shows that, with the PR parameters used to model sleep regulation in humans, the two thresholds in the two-process model are very close, hence the circadian oscillation is the dominant sleep regulator and the two thresholds merge almost into one.

The link between the PR model and the two-process model not only gives us a physiological interpretation of the thresholds in the two-process model, it also allows us to gain a greater insight into the dynamics of the PR model, enabling understanding developed in the context of the two-process model to be interpreted in the physiological setting of the PR model. In the next sections, two different examples are discussed.

### Transitions from monophasic to polyphasic sleep

It is well-known that the two-process model can show a range of different sleep-wake cycles, including cycles that have multiple sleep episodes each day, see Figure 1(a), and cycles that have a period greater than one day, see Figure 1(c). Indeed in [10], the authors postulate that the two-process model can explain the polyphasic sleep of many animals. In [24], it is shown that the sleep-wake cycles of many different mammals can be understood by varying two parameters in the PR model: the homeostatic time constant 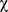 and the constant component to the VLPO drive, 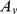. In the previous sections, we have demonstrated how the parameters of the PR model relate to those of the two-process model, specifically, the homeostatic time constant 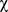 is present in both models and varying the drive to the VLPO 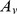 corresponds to varying the upper and lower thresholds without changing the distance between them. In [29], [ 30] it is shown that the two-process model can be understood as a one-dimensional map with discontinuities. In this section, we use this map to show how the observations in [24] and the postulate in [10] are linked and clarify how the transition between different numbers of daily sleep episodes occurs.

**Figure 5:**
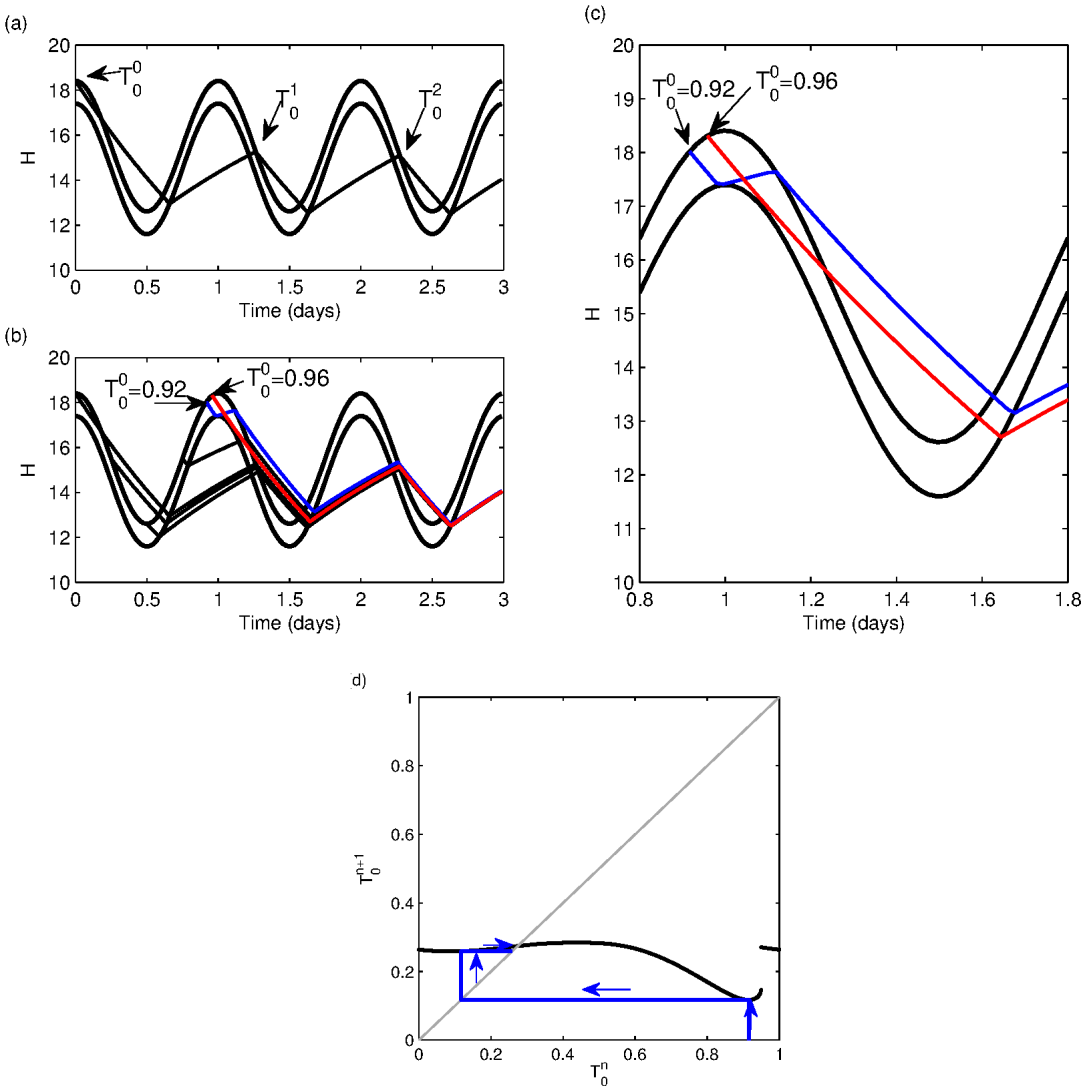
The one-dimensional map for the two-process model. (a) A single trajectory of the two-process model showing successive times of sleep onset. (b) Trajectories of the two-process model for different initial sleep onset times. Each different sleep onset time results in a different sequence, 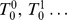, but each sequence rapidly converges to the same sleep onset time, of 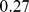 modulo 1 day. (c) A zoom of (b), showing only the trajectories for 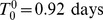 and 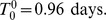 (d) First return map for the two-process model. The black line shows the return map, in other words for any value of sleep onset time on day 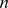, 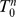 it shows the onset time of sleep on day 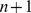, 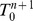. The grey diagonal line is the line along which 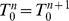. One typical trajectory is plotted for 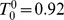 showing the rapid convergence to the periodic cycle where 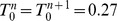 modulo 1 day, the point at which the return map and the diagonal line intersect. The discontinuity is a result of the fact that neighbouring values of 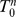 exist that lead to very different values for 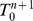, as shown in (c). Parameter values for the two-process model are based on the PR model for the human sleep-wake cycle and can be found in (15).

First we introduce the one-dimensional map. Consider the two-process model and suppose we start on the upper threshold, at time 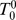, where the model switches from wake to sleep. The dynamics of the two-process model takes this starting point and, propagating it forward through one sleep and one wake episode, results in the next wake to sleep time, 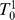, and then through a further sleep-wake episode to 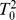 and so on, generating a sequence of sleep onset times 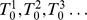. This is illustrated for 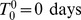 in Figure 5(a). Different starting values 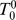 generate different sequences of sleep times, as illustrated in Figure 5(b). For the parameter values chosen here, all sequences converge rapidly to the same monophasic periodic cycle. A graphical way of understanding this sequence is to plot 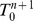 modulo 1 day against 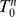 modulo 1 day (the first return map). For any particular starting value, the sequence of iterates can then be found by drawing the cobweb diagram, as shown in Figure 5(d). A monophasic sleep pattern corresponds to 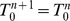 modulo 1 day and so corresponds to the intersection of the diagonal line with the map. The fact that the sequences converge rapidly is related to the fact that the gradient of the map is close to zero for most values of 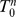. This rapid convergence means that a temporary change to timing of sleep will revert to the regular sleep-wake cycle within a few days.

Phrasing the two-process model in these terms illustrates that it can be represented as a one-dimensional map. Probably the most well-known example of such maps is the logistic map [32] which has been widely used to show that simple rules can lead to very complex dynamics. A distinctive feature of the two-process model is the fact that the map contains a discontinuity. For the parameter values shown in Figure 5(d) this discontinuity occurs at 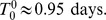 The discontinuity is a consequence of the fact that there exist neighbouring starting values 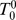 that lead to trajectories that follow very different paths. These occur whenever there are points that result in trajectories that become tangent to the thresholds. For example, starting at 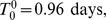 the first sleep just misses the wake threshold at 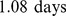 so remains asleep until 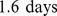 resulting in a sequence 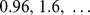, as shown in Figures 5(b) and (c); whereas starting at the nearby value of 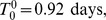 the trajectory hits, rather than misses, the sleep threshold and the resulting sequence is 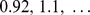.

**Figure 6:**
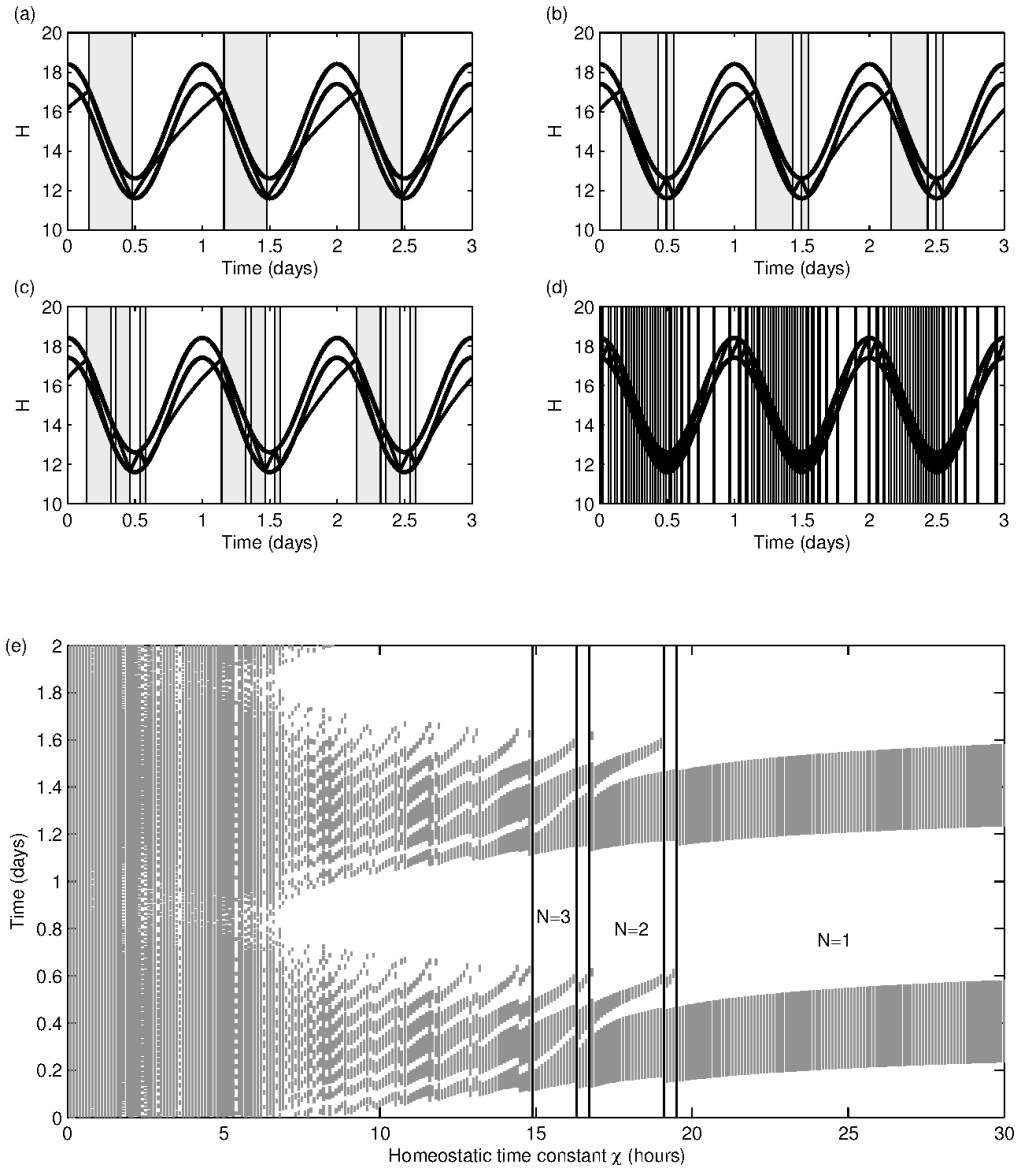
Varying the homeostatic constant. Using the two-process model with parameters as indicated in (15). Figures (a)–(d) give sleep-wake cycles for different values of the homeostatic time constant 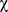 (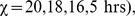 illustrating that reducing 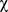 results in more daily sleep episodes. (e) Sleep regions (shaded) as a function of 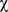. Note that the circadian maximum occurs at 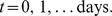

For the value of the clearance parameter 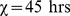 used in Figure 5, the discontinuity does not have a significant impact on the dynamics and all trajectories converge rapidly to the same periodic cycle. However, the presence of the discontinuity is key to understanding the transition from monophasic to polyphasic sleep. This is illustrated in Figure 6(a)–(d), where a sequence of converged solutions to the two-process model are shown for decreasing 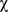. For 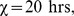 the sleep-wake cycle is monophasic, but in the wake episode the trajectory comes close to, but does not touch, the upper threshold (Figure 6(a)). If distance from the upper threshold is a measure of sleepiness during wake, this would correspond to a dip in alertness. If 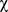 is reduced further, say to 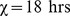 as shown in Figure 6(b), then the wake trajectory does not only come close to, it touches the upper threshold resulting in a short nap and a sleep-wake cycle that is bi-phasic with one longer sleep and one short sleep. Decreasing 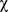 further results in a sequence of further tangencies each of which adds one additional sleep-wake episode. Such transitions are known as grazing bifurcations, tangent bifurcations, or border collision bifurcations and are characteristic of one-dimensional maps with discontinuities [33]–[35]. In the return map, a grazing bifurcation occurs when the discontinuity in the map coincides with the diagonal line. They are responsible for period-adding transitions in the context of electronic circuits and here, we see, are responsible for sleep-episode-adding transitions. Such transitions have also been observed and analysed using one-dimensional maps in the context of understanding the dynamics of neurons [36], [ 37].

**Figure 7:**
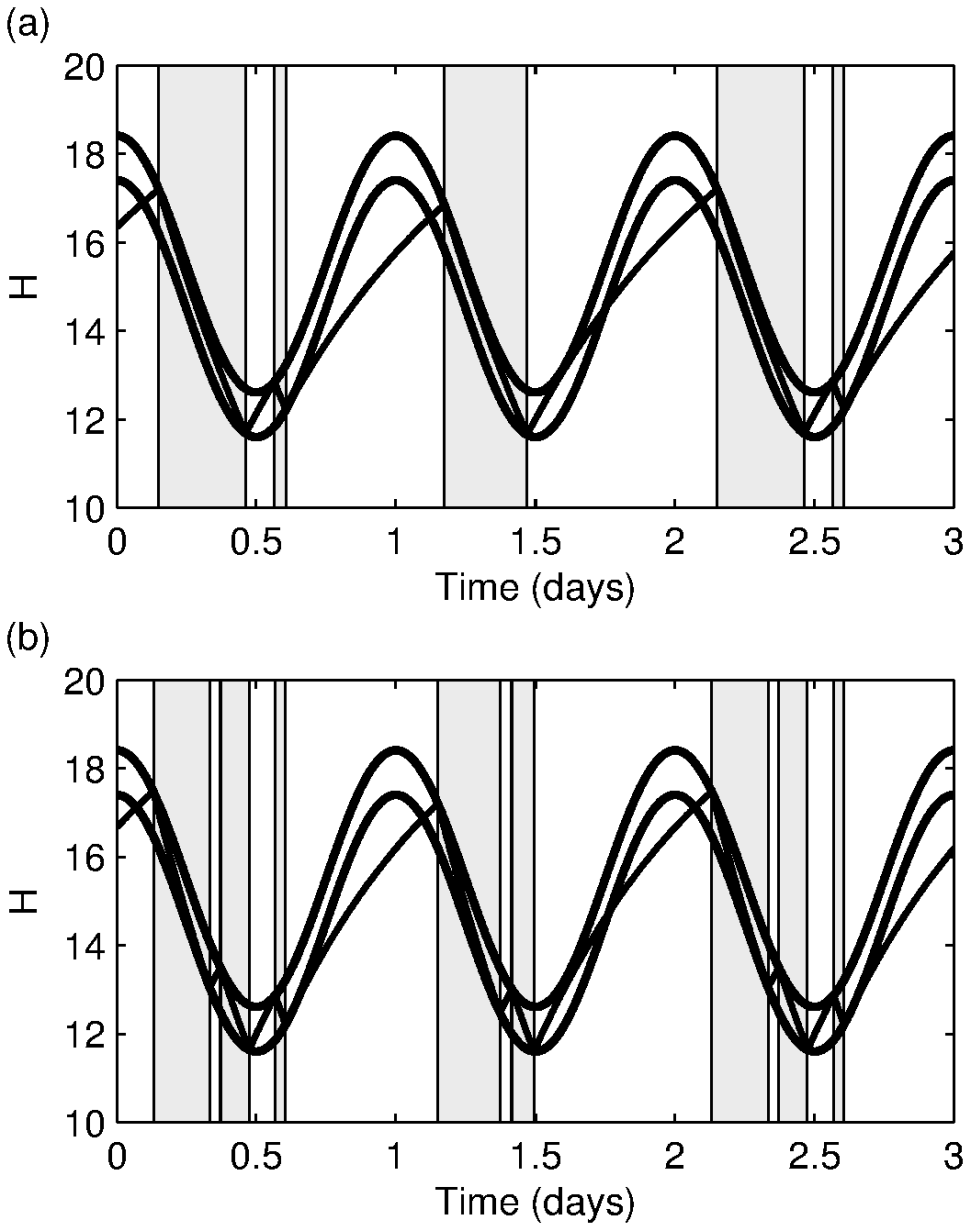
Sleep-wake cycles with a two day period. Solutions of the two-process model showing periodicity on the period of two days. (a) 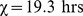 (b) 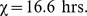 All other parameters are as in Figure 6 and can be found in (15).

The sleep-wake pattern for varying 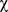 is shown in Figure 6(e). For larger values of 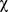 there is one episode of sleep each day: the model falls asleep exactly once and always at roughly the same time (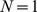). A grazing bifurcation occurs at around 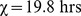 and results in a region between 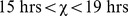 where sleep is bi-phasic with one longer and one shorter sleep each day (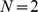). A succession of further grazing bifurcations take place as 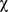 is reduced, resulting in increasing numbers of daily sleep episodes. From Figure 6(e) we see there are intermediate regions between each value of 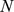. For example, between the monophasic and biphasic region there is a small region around 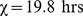 where the sleep pattern has a period of two days. This corresponds to a region where a grazing bifurcation has taken place, causing an extra sleep period on one day, but this extra sleep period is enough to mean that no additional sleep is needed on the following day. The sleep wake trajectory in this case is shown in Figure 7(a). Similar behaviour is seen at each transition between different numbers of daily sleep episodes and is characteristic of such transitions in one-dimensional discontinuous maps [38]: this is illustrated for the transition between two and three sleep episodes in Figures 7(b) and is a similar pattern of sleep to that shown in Figure 1(a) using parameters as in [10]. In fact, as shown for one-dimensional discontinuous maps in [38], the situation is even more complicated: in Figure 1 of [27] the first few layers of an infinite adding scheme are set out. This shows that, for example, the sequence of transitions from sleeping once a day to sleeping twice a day is 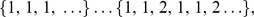 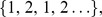 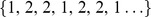 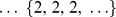, etc. Further discussion of the map is given in the Information S1.

**Figure 8:**
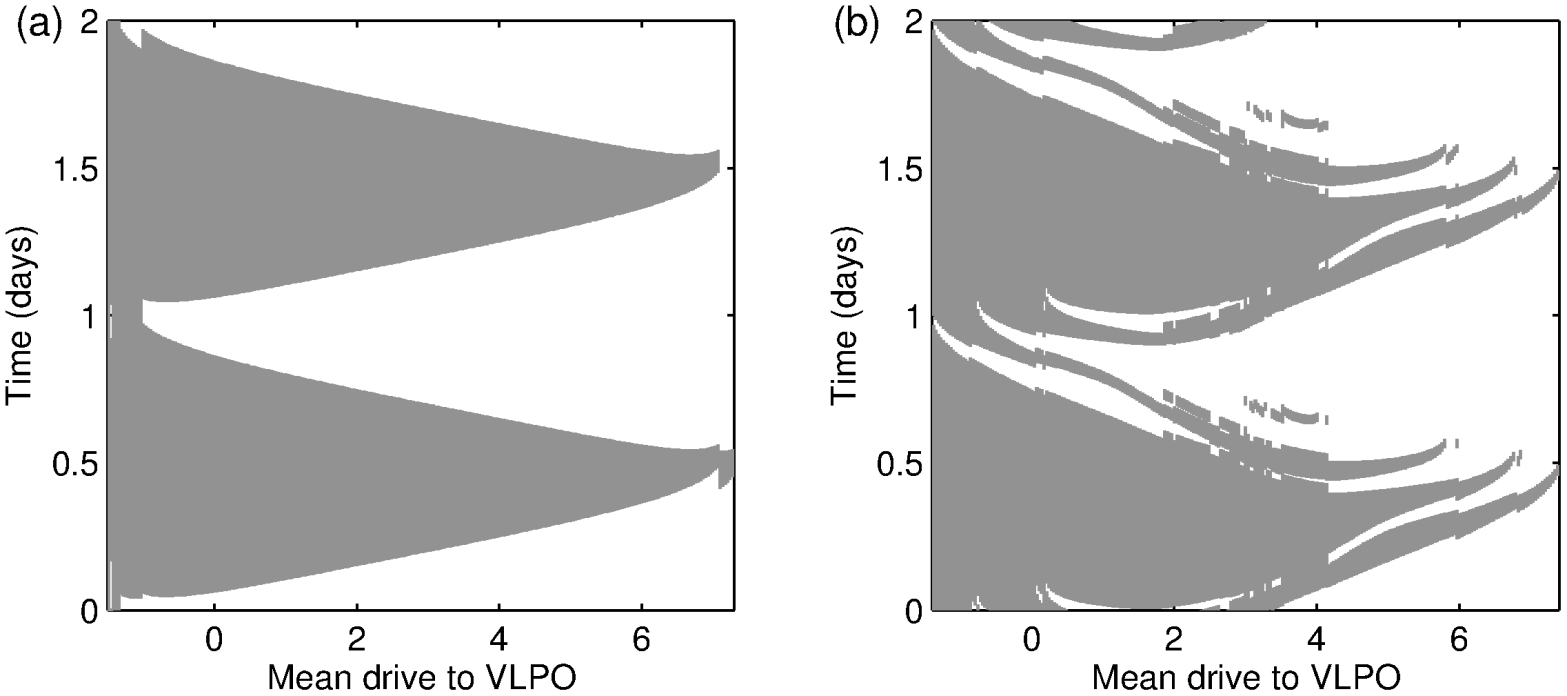
Sleep timing in the two-process model. The upper and lower thresholds are moved simultaneously via 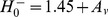 and 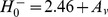 with (a) 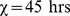 (b) 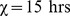 and all other parameters as in (15). Note that mean VLPO drive equals 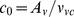 for consistency with [24]. Sleep regions are shaded.

In [24], the behaviour of the PR model is examined both as the time constant 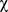 and the mean drive to the VLPO, 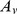 are varied. Our parameter equivalences between the PR model and the two-process model (9) show that increasing 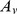 is equivalent to increasing the upper and lower thresholds without changing the distance between them. One can then deduce for the two-process model that for low 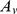, the homeostat will never reach the lower threshold and no wake will occur. Similarly, for high 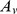 no sleep will occur. For large values of 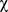 (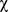 greater than approximately 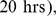 the amount of daily sleep varies approximately linearly with the mean drive to the VLPO as shown in Figure 8(a) and observed in [24]. As seen before, the sleep-wake cycle is monophasic and is largely independent of 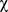 in this range. The actual transition between monophasic sleep and no sleep (or no wake) occurs through grazing bifurcations, where this time the grazing bifurcations result in periodic cycles that have wake (sleep) episodes of greater than 24 hours: examples of such cycles are evident in Figure 8(a) at the extremes of the values of 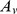 that are shown. For smaller values of 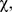 where polyphasic sleep exists, varying 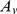 shows that, as the no sleep (or no wake) threshold are approached, grazing bifurcations result in ever decreasing numbers of sleep (wake) episodes until no sleep (no wake) occurs, see Figure 8(b).

In [24], it was shown that the sleep of many mammalian species could be understood in the context of the PR model by varying just two, physiologically plausible, parameters: 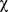 and 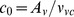. Their results show: a sequence of transitions from monophasic to polyphasic sleep as the time constant 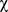 is reduced but where total sleep daily sleep remains approximately constant; for fixed 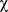 and varying mean drive to the VLPO a sequence of transitions from a state with no wake to a state with no sleep. By using the relationship between the PR model and the two-process model we see that reducing 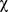 results in a sequence of transitions from monophasic to polyphasic sleep through grazing bifurcations that successively add sleep episodes; at the transition between 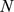 episodes of sleep and 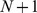 episodes of sleep, there are regions where sleep alternates between 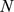 and 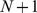 daily episodes (examples of such trajectories for the PR model are shown in Information S1). The identified parameter equivalences show that changing the mean drive to the VLPO is equivalent to simultaneously shifting the upper and lower thresholds of the two-process model. The relation between the PR model and the two-process model shows how this inevitably leads to grazing bifurcations and ultimately cycles with either no sleep or no wake.

The quantitative agreement with [24] is close, but not exact: this is because we have chosen a fixed value for 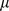, the upper asymptote, in the two-process model, the value to match the PR model for 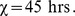 Varying 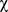 in the PR model results in a small change to the precise region of the switching function that is used, which in turn induces some change in the value of 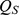. Since 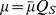 this results in some dependence of 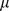 on 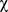 in the equivalent two-process model. One consequence is that the switch from monophasic sleep to biphasic sleep occurs at around 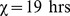 for the two-process model instead of 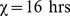 for the PR model. More details can be found in the Information S1.

### Wake effort

Sleep deprivation experiments involve keeping subjects awake for an extended period of time during which cognitive and behavioural tests are undertaken to measure sleepiness and performance. One measure of sleepiness is the Karolinska Sleepiness Scale (KSS) score [39]. In [25], the concept of ‘wake effort’ is introduced for the PR model and good agreement between wake effort and experimental data on KSS scores is found. Wake effort corresponds to a change in the drive to the MA and is interpreted as a need to provide the MA with greater stimulation in order to maintain wake. Here, we show how this can be re-interpreted in the context of the two-process model.

**Figure 9:**
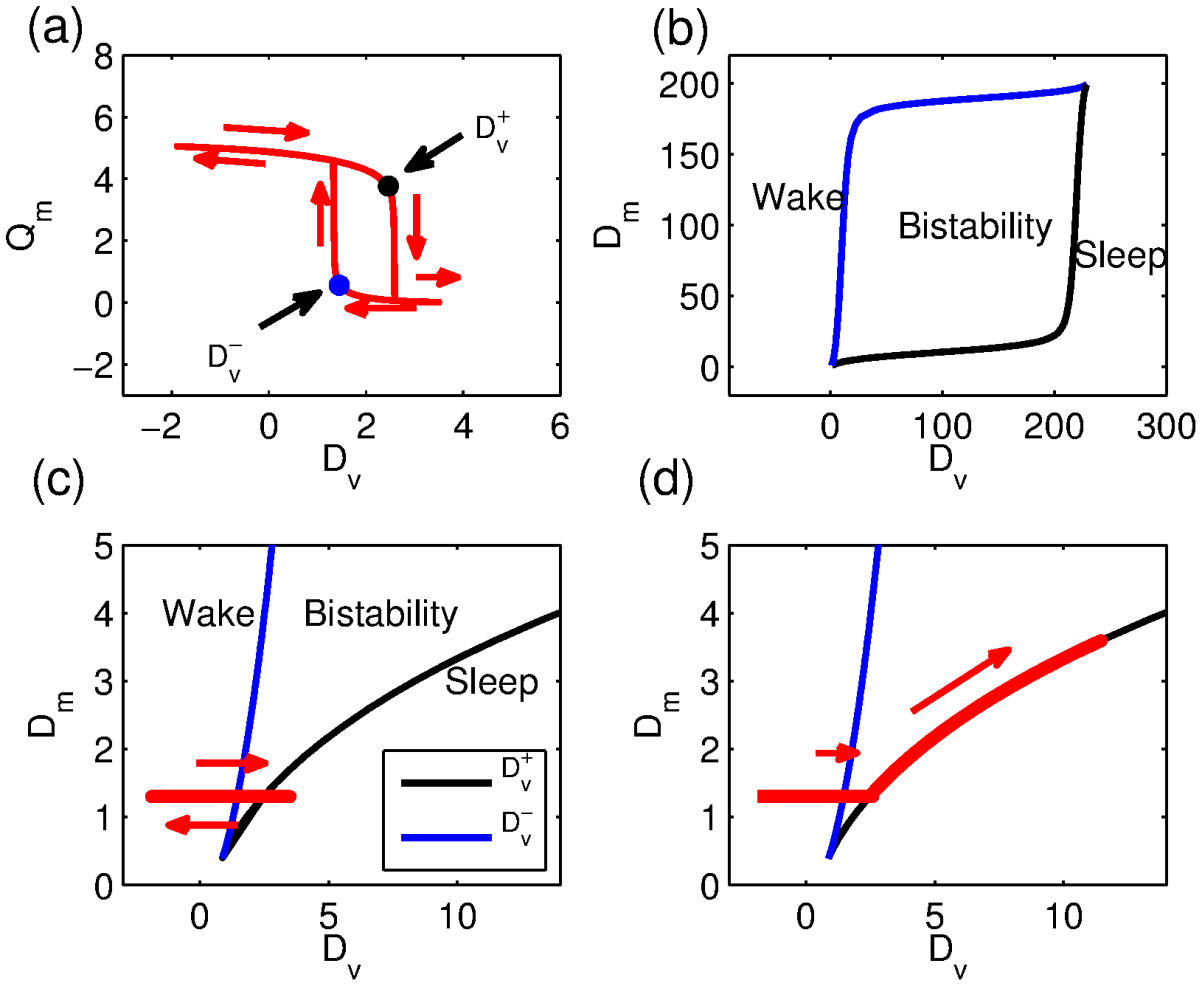
Normal and deprived sleep in the PR model. (a) Sleep-wake cycle showing the MA firing rate 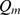 as a function of the drive to the VLPO 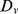. Over one cycle 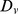 oscillates between high and low values. When 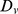 is low, 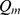 is high and the model is in a wake state. When 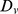 is high, 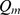 is low and the model is in a sleep state. The transitions from wake to sleep and sleep to wake occur at 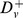 and 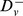 respectively. The size of the hysteresis loop depends on 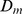, shrinking to nothing for 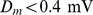 and for 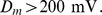 (b) The path of 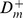 and 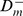 in the 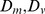 plane. 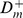 and 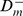 do not exist for values of 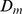 that are either less than 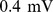 or greater than 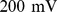. Consequently for 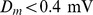 or 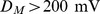 increasing 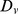 will result in a smooth change from high 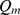 (wake) to low 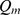 (sleep) instead of the jump from one state to the other shown in (a). (c) A blow up of (b), with the ‘normal’ sleep-wake cycle superimposed. (d) The 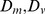 plane showing the wake trajectory in a sleep deprivation experiment.

Wake effort in [25] is presented by considering the graph of the MA firing rate 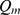 (or equivalently, 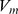), against the drive to the VLPO, 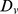. In a regular sleep-wake cycle, 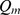 follows a hysteretic loop, see Figure 9(a), where the transition from wake to sleep occurs close to 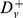 and the transition from sleep to wake occurs close to 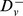. During sleep deprivation, it is argued in [25] that by increasing 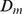, rather than switch from wake to sleep, it is possible to stabilise the ‘ghost’ of the wake state: the extent to which 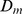 is increased is known as the wake effort. An alternative view of the same idea is to consider the 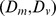-plane as shown in Figure 9(b) and recognise that 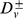 are curves that divide the parameter plane into regions where only the wake state exists, only the sleep state exists, and a bistable region where both wake and sleep exist. There are also regions for low 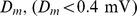 and high 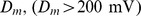, where the two states cannot readily be distinguished. The region of relevance for the parameters used in [25] is close to the bottom of the bistable region, and is shown in blow-up in Figure 9(c). The horizontal line represents the normal sleep-wake cycle: the time dependence of the homeostatic and circadian processes result in 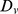 oscillating backwards and forwards along the line, switching from wake to sleep for increasing 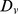 when 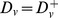 and from sleep to wake for decreasing 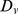 when 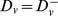.

In sleep deprivation experiments, subjects are prevented from falling asleep at 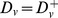. At this point, in order to remain awake the only alternatives that keep the system in the wake region are: decrease the drive to the VLPO, 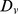; increase the drive to the MA, 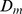 or some combination of both of these. In [25], it is argued that in order to maintain wake it is necessary to stimulate the MA, and therefore 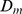 is increased to remain on the ‘ghost state’, but this is equivalent to following the line 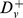. The additional amount by which the MA is stimulated, the wake effort, 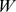 is then 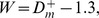



where 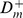 is a function of 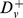 and is the solution of equations (14) in the Methods section.

**Figure 10:**
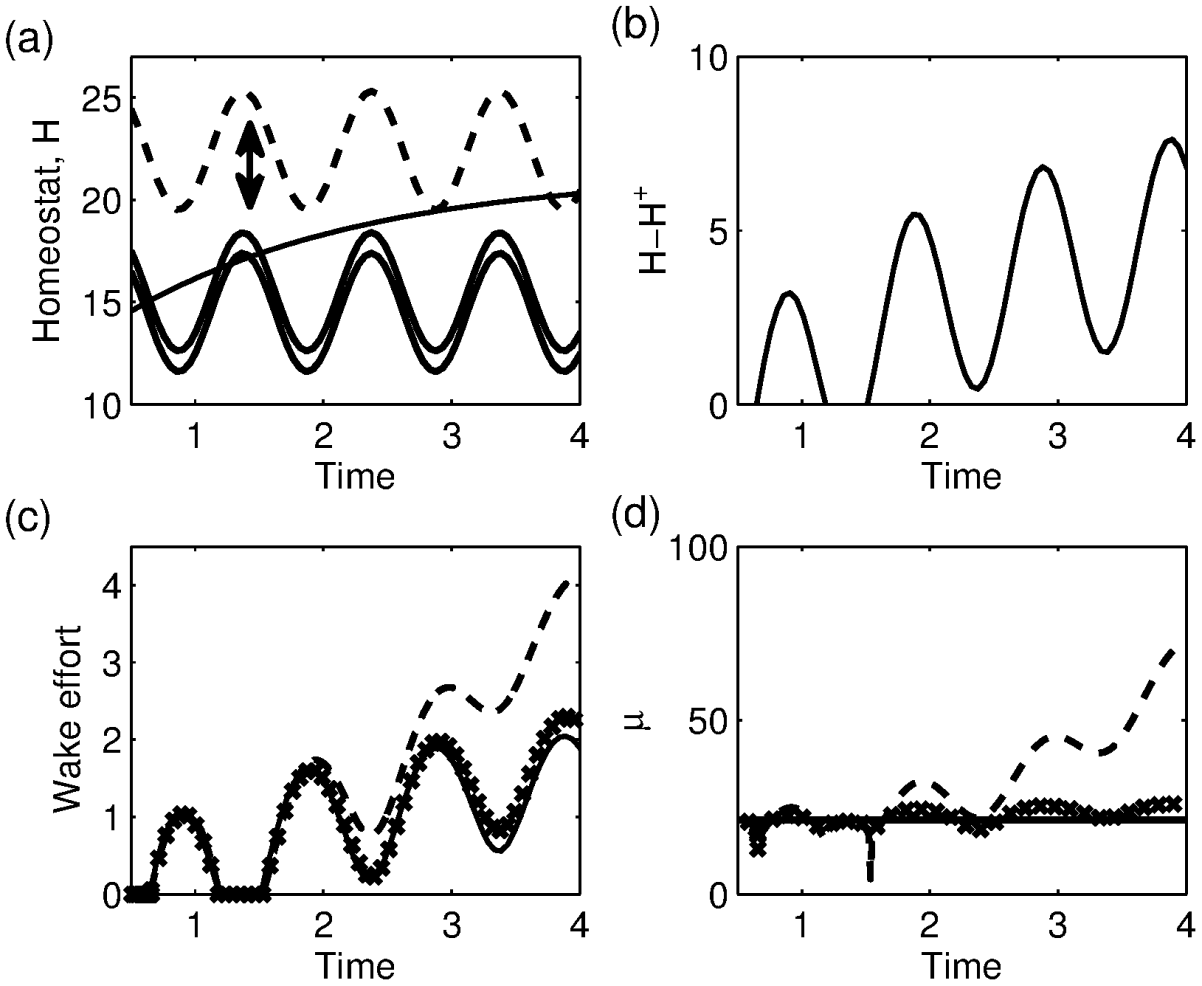
Sleep deprivation and the wake effort. (a) The two-process model, showing the typical trajectory of the homeostatic pressure during a sleep deprivation experiment. Using the wake effort concept of [25] suggests that the upper threshold moves simultaneously: the dashed line shows the position of the upper threshold after 4 days. (b) The difference between the homeostatic pressure and the value at the ‘normal’ threshold, 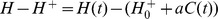. (c) The wake effort computed from the two-process model (10) (solid line), the PR model as in [25] using 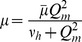 (crosses), the PR model with 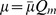 (dashed line). (d) The dependence of 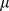, the upper asymptote, on time for the three different cases shown in (c). The downward spikes indicate that the model gets very close to falling asleep, hence 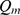 gets very close to 0.

In the two-process model, acute sleep deprivation is modelled as a continued increase in the homeostatic pressure. In [10] this is interpreted as a suspension of the upper threshold, but with insight gained from the the PR model, we see that an alternative interpretation is that the upper threshold is continuously moved to keep the model in the wake state, as shown in Figure 10(a). The wake effort is then related to the extent to which the threshold has to be moved, that is the quantity 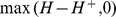 with the upper threshold 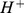 as given by (3). This quantity is shown in Figure 10(b). In the Methods section it is shown that this relation is 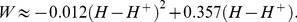
(10)


This resulting wake effort computed from the two-process model is shown by the solid line in Figure 10(c) and agrees very well with the calculation of the wake effort from the PR model in [25] (crosses).

The close to linear relationship (the quadratic term has a very small coefficient) between wake effort in the PR model and 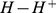, which is essentially the difference between the homeostatic pressure and the circadian oscillator, demonstrates that the wake effort used in [25] is fundamentally similar to previous measures used to compare performance and sleepiness scores. The precise scaling relationship and the degree of nonlinearity is dependent on the shape of the bistable region in the 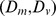-plane shown in Figure 9(b), and on the choice of function for the dependence of the homeostatic process on the firing rate of the MA. In [17] and for many of the subsequent papers, the upper asymptote is give by 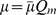. However, in [25] the functional form 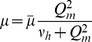 is used in order to “limit the unrealistically high production rate at high 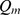
”. This change in functional form has the effect of keeping 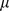 approximately constant during wake, which is why the agreement between the wake effort as defined by [25] agrees well with our analogous computation from the two-process model. This is illustrated in Figure 10(c) and (d) where the wake effort and the dependence of 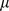 on time are shown for the two-process model and for the PR model with the two different functional forms for 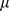.

The shape of the bistable region in the 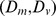 plane shows that for 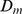 larger than about 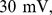 there is a transition from relatively small changes in 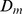 needed to maintain wake to very large changes in 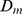 needed to maintain wake; eventually it becomes impossible to maintain wake at all. While for typical parameters used in the PR model this transition occurs for infeasibly large values of 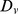 and 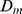, we note that the shape of the bistable region is dependent on the parameters within the firing function and the choice of firing function itself. Once fixed in [31] these parameters have largely been left unchanged: we will return to this point in the discussion.

## Discussion

The strengths of the two-process model have been its inclusion of the two fundamental processes that are believed to regulate the sleep-wake cycle along with its graphical simplicity. This has meant that it has been used extensively as a tool to understand the behaviour of the sleep-wake cycle, design experiments and interpret data [40], [ 41]. A weakness is the difficulty in relating the threshold levels of the homeostatic pressure 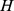 that result in switches between wake and sleep to physiological quantities.

The PR model was developed with the same two governing processes in mind, but introduced some physiological basis for the switching that occurs between wake and sleep. In recent years, this model has been extensively tested in a range of scenarios, some of which depend on the fast dynamics within the model, like the role of disturbances during sleep [23], but in many cases relying on the slow dynamics of the model. The four orders of magnitude between the neuronal timescale and the homeostatic/circadian times scales means that the timescale separation between the slow and fast dynamics is very good.

Here we have shown that the slow dynamics of the PR model can be explicitly related to the two-process model, which provides new perspectives on both the two-process model and the PR model. Using this relationship, new insight into the meaning of the two-process model has been gained. Specifically, the distance between the thresholds is related to the degree to which the MA inhibits the VLPO during wake and the values of the thresholds are related to the parameters associated with the modelling of the firing rates 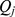, the mean VLPO drive, and the strength of the homeostat. The parameter comparison also highlights the fact that there is no strong reason why the homeostatic pressure should remain between the thresholds in the two-process model, see for example Figure 4. For values between the thresholds, either sleep or wake can occur. Above the upper threshold, only sleep can occur: this could be viewed as a region of sleep, from which it is hard to wake up. Below the lower threshold, only wake can occur, representing times when it is difficult to fall asleep.

Motivated by the strong relationship between the two-process model and the slow dynamics of the PR model, we have re-visited the two-process model in order to gain insight on the dynamics of the PR model. By using the fact that the two-process model can be represented as a one-dimensional map with discontinuities we are able to interpret the transitions from monophasic to polyphasic sleep as grazing bifurcations. This provides the dynamical underpinning for the observation that the PR model gives a systematic framework which encompasses many different mammalian species and confirms the hypothesis of [10] that such a framework could be present in the two-process model. Furthermore, it suggests that ‘typical’ transitions with varying clearance parameter, at least for the larger mammalian species with relatively large clearance parameters, will involve gaining or losing one sleep episode a day. We note that the sequence of transitions for increasing 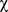 is consistent with observations of changes in the daily sleep patterns of early childhood.

Varying the homeostatic time constant as shown in Figure 6(a) suggests that for large mammals (large 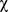) sleep regulation is dominated by the circadian rhythm. In contrast, as shown in Figure 6(d), small mammals are more strongly driven by their metabolism and it is the homeostatic component that dominates. However, we note that the equivalence of the two models raises some interesting questions on accepted parameter values: in both models the homeostatic process is modelled in a similar way, with exponential decay during sleep and an exponential approach to an upper asymptote during wake. In the context of the two process model, accepted physiological markers for the homeostatic process are slow waves in the sleep EEG and theta activity in the EEG during wake respectively, both of which are readily measured. The time constants 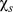 and 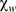 differ during wake and sleep and are measured to be 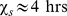 and 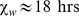 in humans [11]. An important physiological question is the necessity for two different time constants for the homeostatic process, one for wake and one for sleep. Animal [42] and human experiments [43] strongly suggest that the time constant during wakefulness varies with genetic background (animals) and during development (humans) whereas the time constant during sleep appears more invariant within species. In the context of the PR model, the homeostatic process represents the concentration of somnogenic factors such as adenosine, which are not easily accessible. During wake, adenosine is produced more quickly in the brain than it is cleared, decreasing the inhibition to the VLPO. A single value 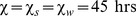 is taken in order to replicate typical sleep patterns for adult humans. Given that in both models, the homeostatic process plays a key role in determining patterns of sleep and wake, it would be interesting to extend the modelling of the homeostatic process in the PR model to allow 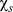 and 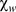 to differ and determine whether a different parameterization of the PR model would lead to time constants in-line with measured values for the two process model.

The grazing bifurcations have been shown to occur as the clearance parameter 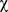 and as the mean drive to the VLPO or equivalently, both the upper and lower thresholds, are simultaneously varied. However, it is clear that the tangencies between the sleep-wake trajectories and the thresholds that give rise to these bifurcations could also occur if the the distance between the thresholds (see [29], [ 30]) or the upper and lower asymptotes of the homeostatic process are varied. A systematic study will be carried out elsewhere.

The two-process model has been compared with sleep deprivation experiments by assuming that the upper threshold is no longer present and that the sleep pressure continues to increase, with sleepiness linearly related to the difference between the homeostat and the circadian process. Here, we have demonstrated that the notion of ‘wake effort’ introduced in [25] is a similar measure and is equivalent to imagining, not that the upper threshold has vanished, but that increasing the stimulation to the MA results in increasing the upper threshold in line with the increase in 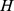.

Similarly, one could also imagine a ‘sleep effort’ that would be required to keep the model asleep when it would naturally wake. This could be achieved by reducing the lower threshold in the two-process model or, equivalently, decreasing the stimulation to the MA, 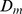. As can be seen from Figure 9(b), the PR model parameters suggest that, while it is possible to extend the wake state significantly by increasing 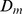, the capacity to extend the sleep state is more restricted. This observation is sensitive to the precise parameters and definition of the firing function. The asymmetry between sleep and wake is equivalent to the fact that in [25], the authors noted that the ‘sleep ghost’ is less prominent than the ‘wake ghost’.

The equivalence between the PR model on the slow timescale and the two-process model is exact when the firing function is a hard switch, but when the firing function is sigmoidal the equivalence is more subtle. This is because, in the PR model, the upper/lower asymptotes of the homeostatic process are modelled as a a function of 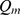 the firing rate of the MA. With a hard switch, 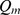 takes only two values, 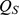 or zero (similar to the two-process model), but with a sigmoid it varies continuously. Except in the neighbourhood of bifurcations, for monophasic sleep we have shown that one can fix the maximal value of 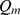 and the switching voltage 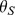 such that the times when the homeostatic pressure reaches its extreme values in the PR and two-process models co-incide. The precise values of 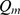 and 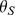 needed, and therefore the values of the asymptotes in the equivalent two-process model, depend to some extent on the other parameters in the model. In this paper we have taken the approach of fixing the values of the asymptotes as those needed to agree with the PR model for their ‘normal’ values of the parameters at 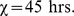 We have not then varied the asymptotes as other parameters are changed which means that the quantitative agreement between the results from the two-process model and the PR model are not exact. Nevertheless, the sequence of transitions and the underlying mechanism through grazing bifurcations carry over between the two models with only minor quantitative differences. In the case of the wake effort, the dependence of the upper asymptote on the firing rate in the PR model means that there is approximately a 10% difference in the wake effort between the two-process and PR models after four days.

However, the fact that implicit in the PR model is a non-constant asymptotic value for the homeostatic process has wider implications. Sleep deprivation experiments tend to show a leveling off of psychomotor vigilance test (PVT) scores over a period of a few days, similar to the levelling off seen in the wake effort shown in Figure 10. In contrast, chronic sleep restriction experiments, where subjects repeatedly are allowed less sleep than they need, tend to show a linear increase in PVT over the timescale of typical experiments. In order to explain this, in [13], Avinash *et al* considered a two-process model but suggested that the upper and lower asymptotes varied with time. This idea was generalised in [14]. Both papers suggest that the time variation occurs through some longer timescale process. We note that within the context of the PR model, during sleep deprivation or chronic sleep restriction the values of the firing function will tend to increase, automatically inducing some time dependence in the values of the asymptotes.

The asymptotes and therefore the wake effort in the PR model are sensitive to the particular choice of the firing function and the functional dependence of the upper asymptote on 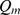. For parameter choices made in [25], 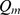, like 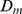, depends approximately linearly on wake effort. However, note that the shape of the relation between 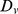 and 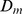 shown in Figure 9 means that for high 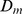 there is a ‘corner’ where to stay awake longer means that a very large increase in 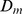 is needed. This transition suggests that a critical change in behaviour for large wake effort, although it is unclear whether this could give an alternative explanation for the behaviour at extreme sleep restriction to the ‘bifurcation’ suggested by [14]. This corner can be further understood by re-examining the firing function shown in Figure 3. Since only a small part of the sigmoid is used under ‘normal’ conditions for the PR model, increasing 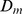 will result in an almost linear change to the range of 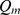. However, once 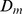 is large, it becomes increasing difficult to increase 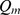 by increasing 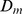 and the corner in Figure 9 corresponds to the flattening off of the relationship between 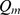 and 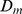. While this is beyond the physiological range of the parameters, this part of the PR model has been less constrained by physiological parameters or behaviour than many other features of the model and a different firing function could lead to a corner at more physiological values. The relationship between the two-process based model in [14], the PR model and the modelling of sleep deprivation versus sleep restriction deserves further attention and will be the subject of a future paper.

In order to better understand sleep/wake regulation it is essential that models that incorporate neurophysiology are developed, analysed and used. However, as models become more complex two problems arise. Firstly they become difficult to analyse systematically, with large numbers of numerical simulations becoming the principle method used to establish the behaviour of the system. Secondly, there is a proliferation of parameters which cannot be easily determined experimentally. One consequence is that it becomes difficult to establish the relative merits of different models. By demonstrating that the two-process model and the PR model are essentially the same for sleep-wake phenomena on the slow time-scale of hours we have not only gained insight on the interpretation of both models but also established the mechanism for transitions between different patterns of sleep and wake in the PR model. This link also suggests some interesting avenues for future extensions of the PR model based on recent insights and research on the two-process and related models.

## Methods

### PR switch to two-process comparison

The equations for the PR switch model are 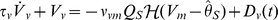


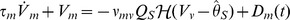


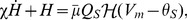
(11)where 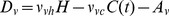


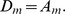



Since 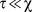 we introduce the small parameter 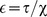, the fast time 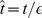 and the slow time 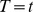, 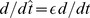 and 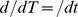. Then, at 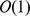 (slow time) equations (11) become 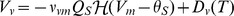


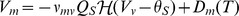


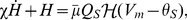
(12)where 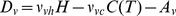


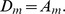



During wake, these have solution 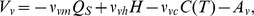


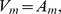


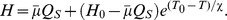



During sleep, these have solution 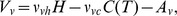


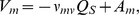


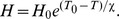



Transitions between wake and sleep when 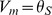, so the switch from wake to sleep occurs when 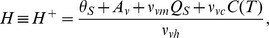
and from sleep to wake when 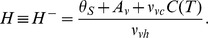



By comparison with equations (1)–(4) we see that the two-process model and the dynamics of the PR switch model on the slow manifold are equivalent if 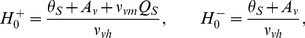
(13)

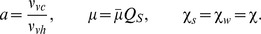



### PR to two-process comparison

On the slow manifold, the PR model is 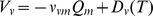


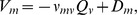


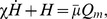
where 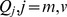 is given by equation (5). For a fixed value of 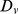 these have one or three solutions, with the transition between one and three solutions happening at saddle-node bifurcations, 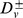 that satisfy 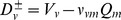


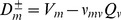
(14)

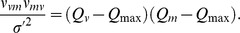



The values of 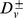 depend on 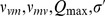 and 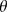, and for the values commonly used in the PR model and listed in Table 1 give 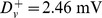 and 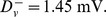


**Table 1:** Typical parameter values for the PR model and the equivalent parameters for the PR model with a hard switch.

Parameter	PR	PR switch
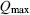 or 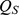	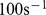	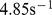
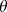	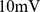	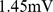
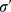	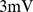	-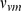	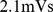	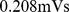
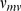	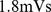	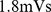
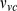	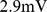	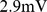
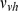	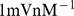	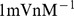
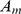	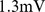	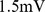
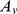	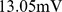	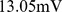
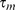	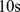	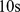
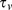	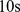	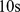
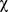	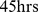	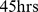
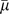	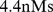	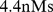

These values give the appropriate parameter values for the two-process model as in (15). The derivation of the values for 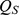, 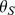 and 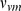 for the PR switch model are given in the Methods section. All parameters have been defined to be positive, consequently some of the signs in equations (6) are opposite to their original definitions in [17]. The mean component of the circadian drive in the PR model has been incorporated in the definition of 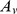, 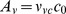, 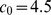.

The sleep-wake cycle corresponds to slowly changing 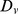, tracing out a path on the slow manifold as shown in Figure 9(a). Transitions from wake to sleep and from sleep to wake occur close to 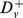 and 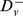 respectively. In order to find parameter values for the two-process model that retain the maximum and minimum values and timings for the homeostatic process for monophasic sleep. Away from bifurcation points the following algorithm is followed:

• First the identification between the threshold values and the saddle node bifurcations in the PR model is made, leading to 
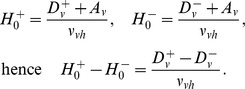



• Numerically integrating the PR model during monophasic sleep results in trajectories for the homeostat that increase to a maximum during wake and decrease to a minimum during sleep, this gives values for 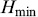, 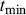, 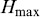 and 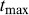. These maximum and minimum values occur close to the switches from wake to sleep and sleep to wake respectively. During wake, the two-process model gives 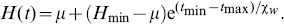



Hence, taking 
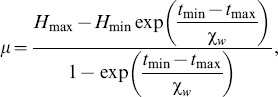
results in a trajectory for the two-process model that passes through the required values at the required times.

• One can do a similar matching for the decreasing 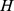 phase to find a value for the lower asymptote. For the simulations presented here, the value of zero was taken for the lower asymptote.

By integrating the PR model with the typical parameter values listed in Table 1, it is found that the minimum occurs at 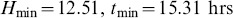 and the maximum at 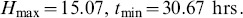 This implies that in this case the parameter values for the two-process model are 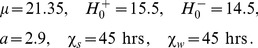
(15)


Going back to the PR switch model and its link to the two-process model, we can now find expressions for the parameters 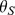 and 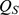 such that it is close to the full PR model:

• Comparing the expression for 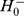 above with that for the PR switch model in (13), gives 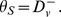



• The relation for 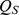 in (13) gives 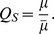



• Considering 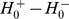 as given above with 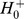 and 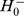 as in (13), leads to 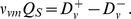



Hence for the typical values of the PR parameters listed in Table 1 and used in Figure 4, 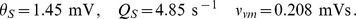



It is also necessary to take 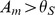 in the PR switch model, otherwise no switching occurs.

### The wake effort

Following [25], the additional amount by which the MA has to stimulated to follow the “wake ghost”, the wake effort, 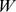 is 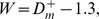
where 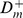 is a function of 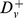 and is the solution of transition equations (14) above. For the region of relevance shown in Figure 9 (c) and (d), the relationship is close to linear with a small quadratic term and is well-approximated by 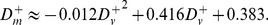



Using the explicit relationships between the parameters in the PR model and the two-process model, the moving of the threshold in the two-process model corresponds to a modified value for 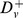 is given by 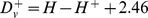, as 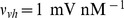 and 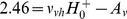, the value of 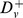 if no wake effort is applied, so the wake effort for the two-process model is 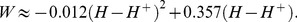
(16)


## Supporting Information

Information S1 (PDF)
