# Supplementary material for: Mathematical Models for Sleep-Wake Dynamics: Comparison of the Two-Process Model and a Mutual Inhibition Neuronal Model
Source: PLoS One. 2014 Aug 1;9(8):e103877. doi: 10.1371/journal.pone.0103877 (PMC4118955; doi:10.1371/journal.pone.0103877)
Supplement: Information S1 — (PDF) [file pone.0103877.s001.pdf]

Mathematical Models for Sleep-Wake Dynamics: Comparison of the Two-Process Model and a Mutual Inhibition Neuronal Model

Supporting Information

Anne C. Skeldon[1,*], Derk-Jan Dijk[2], Gianne Derks[1]
1 Department of Mathematics, University of Surrey, Guildford, Surrey, UK.
2 Faculty of Health and Medical Sciences, University of Surrey, Guildford, Surrey, UK.
∗ E-mail: a.skeldon@surrey.ac.uk

The two-process model as a one-dimensional map

As discussed in the Results section, the two-process model can be represented as a one dimensional map with discontinuities. The map is given by

$$T_0^{n+1} = G(T_0^n) \tag{1}$$

where $G(T_0^n)$ is defined as follows. For $t \in (T_0^n, T_w^n]$

$$H(t) \quad = \quad \left(H_0^+ + aC(T_0^n)\right) \mathrm{e}^{(T_0^n - t)/\chi_s}, \qquad T_w^n = \min(t) \quad \text{s.t.} \quad H(t) = H_0^- + aC(t). \tag{2}$$

For $t \in (T_w^n, T_0^{n+1}]$

$$H(t) \quad = \quad \mu + \left(\left(H_0^- + aC(T_w^n)\right) - \mu\right) \mathrm{e}^{(T_w^n - t)/\chi_w}, \qquad T_0^{n+1} = \min(t) \quad \text{s.t.} \quad H(t) = H_0^+ + aC(t), \tag{3}$$

where $C(t) = \sin(\omega(t - \alpha))$; $H_0^{\pm}$ are the mean values for the upper/lower thresholds; μ is the upper asymptote; χ_s and χ_w are the time constants for the homeostatic process during sleep and wake; a, ω and α are the amplitude, frequency, phase shift, of the circadian process $C(t)$.

In [27] a detailed analysis of piecewise-linear discontinuous maps is presented. It is argued that these represent the normal forms for many systems in the neighbourhood of the discontinuity and that three different bifurcation scenarios are observed in such systems. Of the three scenarios, the particular case that is relevant for the two-process model parameters used to match the PR model in this paper is the scenario labelled as period adding bifurcations. It is not yet clear whether the two other scenarios, which [27] label as period increment with coexistence of attractors and pure period increment scenarios, can also occur.

The sequence of bifurcations shown in Figure 6(e) is typical of period adding bifurcations. A more conventional way to present the bifurcation diagram is shown in Figure S1 where the values of T_0^n are plotted against the bifurcation parameter. The term 'period-adding' refers to the fact that the period of the iterated map changes as a function of the parameter. So, for example, when the number of daily sleep episodes changes from one to two, the map repeats itself after two iterations.

In the main body of the text it is highlighted that between parameter values where solutions with N daily sleep episodes and solutions with $N+1$ daily sleep episodes there are solutions which alternate between N and $N+1$ sleep episodes, as shown in Figure 7. The same sequence occurs in the PR model, as illustrated in Figure S2.

Yet another way of presenting the bifurcation diagram for the iterated map is to plot the length of the daily sleep episodes. This is shown in Figure S3. On this diagram is also plotted the mean daily total sleep. This shows that for the two-process model, as for the PR model, the mean total daily sleep is approximately independent of the homeostatic time constant.

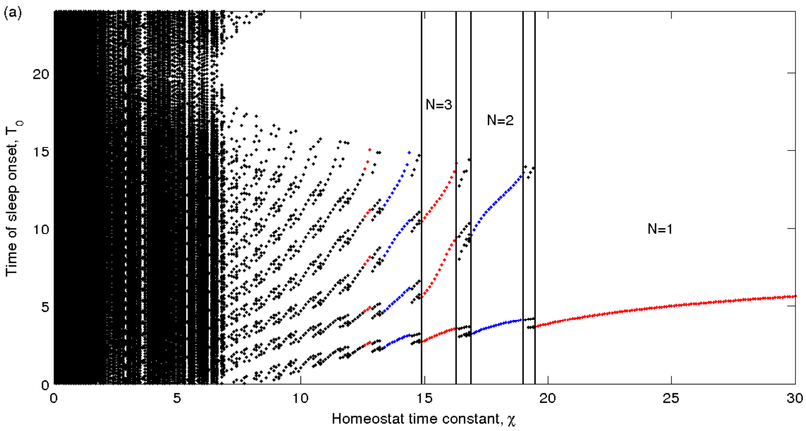

Figure S1: Bifurcation diagram showing the times of sleep onset against the clearance parameter χ in the two-process model. Regions of parameter space where one, two and three daily sleep episodes (N) are delineated but not for higher values for clarity.

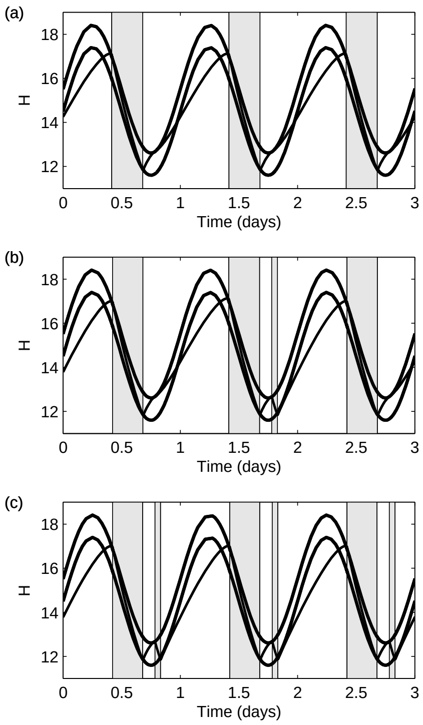

Figure S2: PR model transitions from monophasic to biphasic sleep patterns as χ is reduced. (a) $\chi = 16$ hrs,(b) $\chi = 15.9$ hrs, (c) $\chi = 15.8$ hrs.

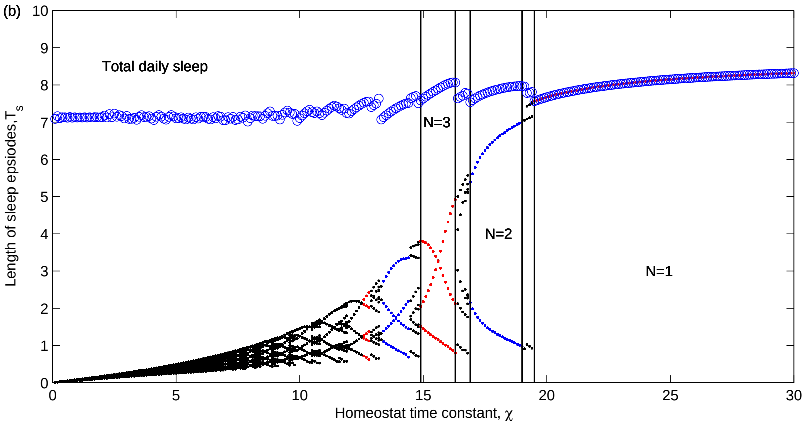

Figure S3: Bifurcation diagram showing length of sleep episode in the two-process model as a function of the parameter χ. So, for example, at $\chi = 22$ hrs there is one single daily sleep episode of length approximately 8 hours and for $\chi = 18$ hrs there are two daily sleep episodes of approximately 6.6 and 1.5 hours respectively.

The upper asymptote

In the Methods section, it is shown how to fit the parameter μ of the two-process model such that the homeostatic switching happens with the same timing as in the PR model. This fitting is dependent on the sleep-wake cycle and if χ varies, this sleep wake cycle will slightly vary and lead to different timings of the homeostat. In its turn, this would lead to a correction for the paremeter μ. In Figure S4 the dependence of the parameter μ is depicted for monophasic sleep.

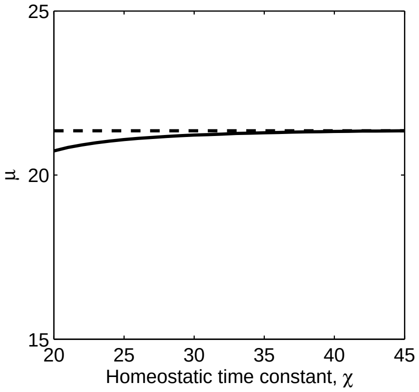

Figure S4: Values of the upper asymptote μ in the two-process model that are needed to fit the PR model for monophasic sleep.
